# The Efficacy, Safety, and Efficiency of the Off-Label Use of Bevacizumab in Patients Diagnosed With Age-Related Macular Degeneration: Protocol for a Systematic Review and Meta-Analysis

**DOI:** 10.2196/38658

**Published:** 2023-06-09

**Authors:** João Estarreja, Priscila Mendes, Carina Silva, Pedro Camacho, Vanessa Mateus

**Affiliations:** 1 Health & Technology Research Center Escola Superior de Tecnologia da Saúde Instituto Politécnico de Lisboa Lisbon Portugal; 2 Centro de Estatística e Aplicações Universidade de Lisboa Lisbon Portugal; 3 iMed.ULisboa Faculdade de Farmacia Universidade de Lisboa Lisbon Portugal

**Keywords:** wet macular degeneration, age-related macular degeneration, AMD, neovascular age-related macular degeneration, nAMD, bevacizumab, drug therapy

## Abstract

**Background:**

Age-related macular degeneration (AMD) is recognized as the leading cause of vision loss in older people. Considering the phenomenon of aging societies worldwide, the prevalence of AMD is expected to increase gradually in the future. AMD can be divided into early, intermediate, and late stages, with the early and intermediate stages being mainly asymptomatic, and the late stage being classified as geographic atrophy, neovascular AMD, or both. Current pharmacological treatments for neovascular AMD include anti–vascular endothelial growth factor agents, such as ranibizumab, pegaptanib, and aflibercept. Additionally, it has been reported that the off-label use of intravitreally administered bevacizumab is effective. It is also lower cost than other agents, which makes it an interesting pharmacological approach.

**Objective:**

This review aims to evaluate the efficacy, safety, and efficiency of bevacizumab for the treatment of neovascular AMD.

**Methods:**

This review will only consider randomized controlled clinical trials that compare the use of bevacizumab with another pharmacological agent or a placebo in patients aged 50 years and older who are diagnosed with vascular AMD. It will exclude studies that include participants diagnosed with polypoidal choroidal vasculopathy or retinal angiomatous proliferation. To identify and select relevant articles, we will develop a highly sensitive search strategy and apply it in MEDLINE via the PubMed platform. Upon selection of the studies and analysis of the titles, abstracts, and full texts, the results will be presented according to the Preferred Reporting Items of Systematic Reviews and Meta-Analyses (PRISMA) guidelines. The analysis and extraction of the data will be performed by 2 independent reviewers. Risk of bias will be evaluated with the Critical Appraisal Skills Programme (CASP) checklist. Finally, the same reviewers will also perform a quality assessment of the included studies with the Grading of Recommendations, Assessment, Development, and Evaluations (GRADE) tool.

**Results:**

The search strategy, after the application of the inclusion and exclusion criteria, identified 15 randomized clinical trials, which are currently being analyzed. This project has no funding and it has been developed by a multidisciplinary research team of pharmacologists and orthoptists. The study was initiated in May 2021 and it is expected to conclude by the end of 2023.

**Conclusions:**

This review will provide a synthesis of current information and underlying evidence about the off-label use of bevacizumab in neovascular AMD. It will provide a clearer vision of a possible new pharmacological approach, as well as the most suitable treatment designs, for the treatment of neovascular AMD.

**Trial Registration:**

PROSPERO CRD42021244931; https://tinyurl.com/p6m5ycpk

**International Registered Report Identifier (IRRID):**

DERR1-10.2196/38658

## Introduction

Age-related macular degeneration (AMD) is closely associated with visual impairment and is considered the leading cause of vision loss in elderly people [1-3]. Indeed, it is reported that 9% of all cases of blindness are related to the progression of this disease [4]. AMD frequency increases with age, and the disease is known to be progressive and degenerative [1,5]. It is estimated that around 200 million people are affected by AMD, and in aging societies, the prevalence of this disease may increase in the near future [3-6]. In addition to age, there are other risk factors for AMD, such as environmental factors, obesity, atherosclerosis, smoking, genetic background, and metabolic and functional factors [2,7].

AMD can be divided into early, intermediate, and late stages. The early and intermediate stages are mainly asymptomatic and represent about 90% of cases [6-8]. Late AMD is classified into 2 main types: dry (geographic atrophy) AMD and neovascular (wet or exudative) AMD. Late AMD is the main cause of AMD-associated visual impairment [2,4,5,8]. AMD diagnosis and grading are based on color fundus examinations in people aged 50 years and older [9]. There are some differences to be aware of between the 2 main types of AMD. Nonadvanced AMD is characterized by typical whitish-yellow focal drusen localized between the retinal pigment epithelium and the Bruch membrane (BrMb). On the other hand, wet AMD is known to induce new abnormal vessels in the macula, resulting in neovascular AMD (nAMD) [10].

AMD pathogenesis is not yet fully understood, but there is already evidence to elucidate the process underlying the onset and progression of this disease [8,10,11]. Both atrophic AMD and nAMD are related to the dysfunction or death of all constituents of the photoreceptor–retinal pigment epithelium–(RPE)–BrMb–choriocapillaris complex, as these constituents work in combination. The development of each type of AMD seems to be associated with the location where the initiating events occur [11]. Atrophic AMD development is characterized initially by the formation of large, confluent drusen and hyperpigmentation, which can be accompanied by RPE dysfunction, leading to risk of geographic atrophy [11,12]. On the other hand, neovascular AMD is associated with the initial loss of choroidal vasculature, which affects the photoreceptor-RPE-BrMb-choriocapillaris complex and appears to be caused by reduced blood supply due to stenosis of the large vessels. An inflammatory environment is established by the accumulation of proinflammatory cytokines, promoting the progression of AMD [11,13]. The RPE remains intact due to the stenosis in the large vessels, but becomes hypoxic and starts to release angiogenic substances, including vascular endothelial growth factor (VEGF), which stimulates the formation of new vessels in the choriocapillaris; this is known as choroidal neovascularization [11].

Because of the process underlying the development of nAMD, current pharmacological treatments are characterized by the intravitreal administration of molecules specifically targeting VEGF [4,14]. The drugs currently accepted by the regulatory authorities for the treatment of this condition include pegaptanib, ranibizumab, aflibercept, and brolucizumab [4,8]. The off-label use of bevacizumab has also been reported for the treatment of nAMD. Bevacizumab is a drug commonly used in oncology for the treatment of metastatic colorectal cancer, breast cancer, and lung cancer [2,4,14]. Indeed, some authors have reported that the intravitreal administration of bevacizumab has a positive influence in cases of nAMD; furthermore, it has a lower cost for the community in comparison with currently used drugs, such as ranibizumab [2,14,15]. Therefore, given the rise in bevacizumab use in nAMD, it would be interesting to specifically evaluate its long-term influence on the onset and progression of this disease. Thus, this project aims to undertake a systematic review and meta-analysis of the evidence concerning the off-label use profile of bevacizumab in the treatment of nAMD through the identification and critical appraisal of clinical studies. One of our specific objectives is to summarize and analyze the current evidence on the clinical outcomes regarding the efficacy, safety, and efficiency of bevacizumab treatment for nAMD. In addition, considering the lack of evidence from current reviews regarding different treatment designs and their influence on outcomes, this review will also aim to fully describe different regimens and determine if there are significant differences between them.

## Methods

### Research Question

The research group follows the recommendations of the Preferred Reporting Items for Systematic Reviews and Meta-Analysis–Protocols (PRISMA-P) for the elaboration of this protocol [16]. This study proposes to answer the following research question: Is bevacizumab more efficient than other pharmacological approaches while having a comparable efficacy and safety profile for the treatment of patients diagnosed with nAMD?

### Participants

In accord with the natural history of AMD and the American Academy of Ophthalmology Preferred Practice Patterns, the study will only include participants diagnosed with nAMD aged 50 years or older [17]. Textbox 1 shows the eligibility criteria.

**Eligibility criteria.**

**Inclusion criteria**

- Participants diagnosed with neovascular age-related macular degeneration and aged 50 years or older
- Treatment with bevacizumab and another pharmacological agent or placebo
- Presentation of outcomes related to the efficacy, safety, and efficiency of the treatment
- Randomized clinical trial

**Exclusion criteria**

- Participants diagnosed with polypoidal choroidal vasculopathy or retinal angiomatous proliferation
- Treatment switched during the study
- Studies written in a language other than English
- Studies published before 2010

### Intervention and Comparison

Patients diagnosed with nAMD and treated with intravitreal bevacizumab will be compared to patients who receive other accepted treatments, such as other anti-VEGF drugs (eg, ranibizumab), or to those who receive a placebo.

### Outcomes

We will analyze several outcomes to evaluate the influence of intravitreal bevacizumab in nAMD in comparison with other established treatments or placebo; these outcomes are related to the efficacy, safety, and efficiency profiles.

### Study Design

This review will only consider randomized controlled trials.

### Search Strategy

This review will use MEDLINE, via the PubMed platform, to develop a highly sensitive search strategy to identify and select relevant and eligible studies. Multimedia Appendix 1 presents the search strategy developed for the MEDLINE database.

### Selection Process

Titles and abstracts of retrieved studies will be screened, and the full text of potentially eligible articles will be assessed by 2 independent reviewers. In case of discrepancies, a third reviewer will be included to make a final decision. Inclusion and exclusion criteria will be applied throughout this process to decide whether a study is included or excluded. Relevant data will be extracted from the included studies and inserted in a customized data extraction document by the 2 independent reviewers. The search results will be presented in the final systematic review and presented as a PRISMA flow diagram. In addition, the reasons for excluding studies will be organized and will also be presented in the final flow diagram. As previously mentioned, we will only include articles written in English and published after 2010.

### Data Collection Process

The retrieved studies from the MEDLINE database will be exported and analyzed through a systematic reviews web application (Rayyan; Qatar Computing Research Institute), which will be used throughout the review for study screening and overall management. The data will be extracted by the same 2 independent reviewers and will include specific parameters concerning the population, intervention, and comparison, as well as outcomes related to the efficacy, safety, and efficiency profiles of the treatments applied. Once again, in case of conflict between the reviewers, a third reviewer will be included to make a final decision. The included studies will be descriptively analyzed and presented in a tabular format. Additionally, the studies excluded from the quantitative synthesis will be subjected to a qualitative analysis in order to gather all available important data. For methodologically and statistically homogeneous studies, we will perform meta-analyses using random effects models to quantify a pooled estimate of the effect of systemic versus ocular adverse events. Meta-analyses will be undertaken separately for each specific study design. We will also consider other subgroup analyses, for instance, of age groups (ie, 50-70 years and >70 years). We will also quantify heterogeneity of the studies using the *I*^2^ statistic and the Kendall τ rank correlation if high values are found; to try to explain heterogeneity across studies, a meta-regression will be conducted. This technique can be performed if there is a suspected variable that may lead to differences in treatment effects across studies. For meta-analysis, we will use the R (version 4.0.5; R Foundation for Statistical Computing) packages *metafor* and *meta*.

### Data Items

During the process of data collection, we will consider several parameters that we will extract and organize, including efficacy outcomes (visual acuity, presence of neovascularization, and retinal thickness), safety outcomes (presence of macular atrophy, endophthalmitis, leakage, and increased intraocular pressure), and efficiency outcomes (administered dose, number of administrations, frequency of administration, duration of treatment, and cost versus benefits of the treatment compared with ranibizumab or placebo).

### Risk of Bias Assessment

All the selected studies will be assessed to evaluate their methodological quality and potential for risk of bias by the same 2 independent reviewers. In case of discrepancies between these 2 reviewers, an additional reviewer will be included to make a final decision. To evaluate the quality and risk of bias in experimental studies, the Critical Appraisal Skills Programme (CASP) randomized controlled trials checklist will be used. We will derive domain-specific and overall quality grading for each study as follows: low risk of bias (A), moderate risk of bias (B), and high risk of bias (C). In addition, we will evaluate the potential of publication bias with funnel plots and the Begg test and Egger test with the trim and fill approach to explore the possible influence of publication bias on the results.

### Registration and Reporting

The protocol for this systematic review was submitted and registered in 2021 in PROSPERO (CRD42021244931). This protocol follows the PRISMA-P guidelines for the development of systematic reviews and meta-analysis. In case there are modifications to the protocol, they will be reported in the final paper with the rationale behind the origin of such amendments.

## Results

The study has included 15 randomized controlled clinical trials after applying the inclusion and exclusion criteria, and the data of interest are being thoroughly extracted. A multidisciplinary research team of pharmacologists and orthoptists has been working on the review since May 2021, as well as on the delineation of the protocol, and it is expected that the final results will be released by the end of 2023.

## Discussion

AMD is closely associated with blindness and has a significant impact on social, health, and economic factors in society. Currently, this disease has no cure and pharmacological treatment aims to slow its rate of progression. Current drug therapies that are accepted for the treatment of nAMD are costly, and bevacizumab may be a possible alternative treatment. The off-label use of bevacizumab in the treatment of nAMD was first reported in 2006, and since then an increase in its use has been demonstrated [2]. Indeed, this drug is now associated with treatment for this disease, taking into account that its efficacy is noninferior compared to ranibizumab, a well-known drug used in this context [18]. According to some of the data being evaluated at the moment, it is already possible to affirm that bevacizumab demonstrates noninferiority compared to ranibizumab and that both drugs need a similar number of administrations to have a comparable effect on outcomes related to efficacy [19,20].

Considering the latest reviews on this topic, it is possible to observe that there is already sufficient information regarding the efficacy and safety profiles of bevacizumab in comparison to other pharmacological approaches [21,22]. However, it is also essential to understand the efficiency of bevacizumab compared with other options, since current approaches are relatively costly, and continuous administration is necessary to slow the rate of progression of the nAMD. After understanding the cost-benefit profile of bevacizumab in comparison with other established treatment molecules, it will be possible to make clinical decisions more effectively, benefiting both the health system and patients.

Thus, the findings from this review will allow a clearer vision of the use of bevacizumab in the treatment of nAMD, as well as its efficacy, safety, and efficiency compared to other drugs and to placebo. Additionally, this key strengths of this study will include synthesizing the information available about the off-label use of bevacizumab in patients diagnosed with nAMD; clarifying the current evidence on the off-label use of bevacizumab in the nAMD context for its efficacy, safety, and efficiency; and developing a comprehensive and highly sensitive search strategy, which will allow the identification of current papers and their underlying evidence. Based on the currently available literature, we are in a position to objectively evaluate bevacizumab for the treatment of nAMD in comparison to other drugs accepted by the respective regulatory authorities.

## Appendix

Multimedia Appendix 1 Search strategy for the MEDLINE database.

## Abbreviations

AMD: age-related macular degeneration
BrMb: Bruch membrane
CASP: Critical Appraisal Skills Programme
nAMD: neovascular age-related macular degeneration
PRISMA-P: Preferred Reporting Items for Systematic Reviews and Meta-Analysis–Protocols
RPE: retinal pigment epithelium
VEGF: vascular endothelial growth factor
