# Correction: The Efficacy, Safety, and Efficiency of the Off-Label Use of Bevacizumab in Patients Diagnosed With Age-Related Macular Degeneration: Protocol for a Systematic Review and Meta-Analysis

**DOI:** 10.2196/50411

**Published:** 2023-07-04

**Authors:** João Estarreja, Priscila Mendes, Carina Silva, Pedro Camacho, Vanessa Mateus

**Affiliations:** 1 Health & Technology Research Center Escola Superior de Tecnologia da Saúde Instituto Politécnico de Lisboa Lisbon Portugal; 2 Centro de Estatística e Aplicações, Universidade de Lisboa Lisbon Portugal; 3 iMed.ULisboa, Faculdade de Farmacia, Universidade de Lisboa Lisbon Portugal

In “The Efficacy, Safety, and Efficiency of the Off-Label Use of Bevacizumab in Patients Diagnosed With Age-Related Macular Degeneration: Protocol for a Systematic Review and Meta-Analysis” (JMIR Res Protoc 2023;12:e38658) the authors noted a missing Acknowledgments section. The Acknowledgments section has now been added to the published paper as follows:

H&TRC authors gratefully acknowledge the FCT/MCTES national support through the UIDB/05608/2020 and UIDP/05608/2020.

The correction will appear in the online version of the paper on the JMIR Publications website on July 4, 2023, together with the publication of this correction notice. Because this was made after submission to PubMed, PubMed Central, and other full-text repositories, the corrected article has also been resubmitted to those repositories.

