# Supplementary material for: The Efficacy, Safety, and Efficiency of the Off-Label Use of Bevacizumab in Patients Diagnosed With Age-Related Macular Degeneration: Protocol for a Systematic Review and Meta-Analysis
Source: JMIR Res Protoc. 2023 Jun 9;12:e38658. doi: 10.2196/38658 (PMC10337418; doi:10.2196/38658)
Supplement: Multimedia Appendix 1 [file resprot_v12i1e38658_app1.docx]

**Supplementary Material 1**

**Search expression:**

((("Middle Aged"[Mesh] OR “Middle Aged”[tiab] OR “Middle Aged” OR "Aged"[Mesh] OR Aged[tiab] OR Aged OR Elderly[tiab] OR Elderly OR Elder) AND ("Wet Macular Degeneration"[Mesh] OR “Wet Macular Degeneration”[tiab] OR “Wet Macular Degeneration” OR “Macular degeneration”[tiab] OR “Macular degeneration” OR AMD[tiab] OR “Neovascular Age-Related Macular Degeneration”[tiab] OR “Neovascular Age-Related Macular Degeneration” OR “Age-Related Macular Degeneration”[tiab] OR “Age-Related Macular Degeneration”)) AND (“Bevacizumab”[Mesh] OR Bevacizumab[tiab] OR Bevacizumab))
